# Designing and implementing a research integrity promotion plan: Recommendations for research funders

**DOI:** 10.1371/journal.pbio.3001773

**Published:** 2022-08-19

**Authors:** Serge P. J. M. Horbach, Lex M. Bouter, George Gaskell, Maura Hiney, Panagiotis Kavouras, Niels Mejlgaard, Nick Allum, Noémie Aubert Bonn, Anna-Kathrine Bendtsen, Costas A. Charitidis, Nik Claesen, Kris Dierickx, Anna Domaradzka, Andrea Reyes Elizondo, Nicole Föger, Wolfgang Kaltenbrunner, Teodora Konach, Krishma Labib, Ana Marušić, Daniel Pizzolato, Tine Ravn, Rea Roje, Mads P. Sørensen, Borana Taraj, Giuseppe A. Veltri, Joeri K. Tijdink

**Affiliations:** 1 Aarhus University, Aarhus, Denmark; 2 Vrije Universiteit Amsterdam, Amsterdam, the Netherlands; 3 London School of Economics and Political Science, London, United Kingdom; 4 Health Research Board, Dublin, Ireland; 5 National Technical University of Athens, Athens, Greece; 6 University of Essex, Colchester, United Kingdom; 7 Department of Ethics, Law and Humanities, Amsterdam University Medical Centers, Amsterdam, the Netherlands; 8 European Association of Research Managers and Administrators, Brussels, Belgium; 9 KU Leuven, Leuven, Belgium; 10 Robert Zajonc Institute for Social Studies, University of Warsaw, Warsaw, Poland; 11 Centre for Science an Technology Studies, Leiden University, Leiden, the Netherlands; 12 Austrian Agency for Research Integrity, Vienna, Austria; 13 University of Split School of Medicine, Split, Croatia; 14 Department of Research in Biomedicine and Health, University of Split School of Medicine, Split, Croatia; 15 University of Trento, Trento, Italy

## Abstract

Various stakeholders in science have put research integrity high on their agenda. Among them, research funders are prominently placed to foster research integrity by requiring that the organizations and individual researchers they support make an explicit commitment to research integrity. Moreover, funders need to adopt appropriate research integrity practices themselves. To facilitate this, we recommend that funders develop and implement a Research Integrity Promotion Plan (RIPP). This Consensus View offers a range of examples of how funders are already promoting research integrity, distills 6 core topics that funders should cover in a RIPP, and provides guidelines on how to develop and implement a RIPP. We believe that the 6 core topics we put forward will guide funders towards strengthening research integrity policy in their organization and guide the researchers and research organizations they fund.

## Abstract 2

Research funders are prominently placed to foster research integrity by requiring that researchers make an explicit commitment to research integrity. This Consensus View suggests 6 core topics that funders should cover in a research integrity promotion plan and provides practical recommendations for how to implement one.

To improve research quality and validity, foster responsible research cultures, and maintain public trust in science, various stakeholders have put research integrity high on their agenda. Among them, research funders are increasingly acknowledging their pivotal role in contributing to a culture of research integrity. For example, the European Commission (EC) is mandating research organizations receiving funding from the €95 billion Horizon Europe program to have, at the institutional level, policies and processes in place for research integrity covering the promotion of good practice, prevention of misconduct and questionable practices, and procedures to deal with breaches of research integrity [1]. To meet these obligations, the EC requires beneficiaries to respect the principles of research integrity as set out in the European Code of Conduct for Research Integrity (ECoC) and suggests that research organizations develop and implement a Research Integrity Promotion Plan (RIPP) [2]. In this Consensus View, we have adopted the World Conference on Research Integrity’s approach to research integrity, by having “research integrity” refer to “the principles and standards that have the purpose to ensure validity and trustworthiness of research” [3]. More specifically, we mostly adhere to the principles outlined in the ECoC: reliability, honesty, respect, and accountability. While many definitions of research integrity exist [4,5], for example, those that distinguish between the integrity of a researcher, integrity of research, and integrity of the research record, the ECoC combines these approaches in a balanced way [1].

We believe that funders are prominently placed to foster a culture of research integrity by requiring that the organizations and individual researchers they support make an explicit commitment to research integrity. At the same time, funders need to adopt appropriate research integrity practices themselves. Of late, attention to research integrity among funders has gathered pace, as reflected in several initiatives around the globe that demonstrate how funders can support a culture of research integrity. For example, the US National Science Foundation (NSF) [6] requires applicants’ research organizations to provide training and oversight in the responsible conduct of research, designate individuals responsible for research integrity, and have an institutional certification to testify of its commitment. Also, in 2016, 3 Canadian federal funders joined forces to support research integrity in the Canadian Tri-Agency Framework: Responsible Conduct of Research–Harmony and Responsibility [7]. The framework was subsequently updated in 2021. This framework sets out responsible practices that research organizations and researchers should follow, including rigor, record keeping, accurate referencing, responsible authorship, and the management of conflicts of interest. It also acknowledges the responsibilities of the funders, including “helping to promote the responsible conduct of research and to assist individuals and institutions with the interpretation or implementation of this Responsible Conduct of Research (RCR) Framework”.

It is not only major funding organizations in highly developed research environments that are taking steps. Smaller funders are also acting to mandate compliance with research integrity standards. The constantly growing literature on the topic is another sign of development within this area [2,3]. In the USA, research integrity recently reached the political arena, when, following a call from researchers [8], President Biden’s administration published a memorandum on restoring trust [9] that highlights the importance of integrity in research. The memorandum will be supported by the reintroduction of the Scientific Integrity Act. This act will prohibit research misconduct and the manipulation of research findings. It talks of a “culture of research integrity” and demands that funding agencies adopt and enforce research integrity policies, appoint a research integrity officer, and provide regular research integrity and ethics training. The US are not alone in their endeavors. Governments in other countries are equally gearing up to support the integrity and reproducibility of research [10]. However, so far, there is only limited evidence about the effectiveness of such initiatives, although it is generally accepted that they raise awareness among various stakeholders concerning research integrity challenges, strengthen the sense of responsibility of those stakeholders to address those challenges, and thereby ultimately contribute to fostering a culture of research integrity.

In a collective effort to foster research integrity, research organizations and funders have their own, complementary roles. The Standard Operating Procedures for Research Integrity (SOPs4RI) consortium has recommended that both research organizations and funders develop a RIPP. A RIPP outlines the key responsibilities of an organization concerning research integrity and details methods and procedures to foster it. For example, in the case of research organizations, a RIPP should facilitate and stimulate a healthy research environment, proper mentoring and supervision, research ethics structures, research integrity training, high-quality dissemination practices, research collaboration, effective data management, and open and fair procedures to deal with breaches of research integrity [11]. Funders have a different role. They can support, safeguard, and incentivise, or even mandate, responsible research practices from research organizations and researchers. Equally important, funders should make sure that their internal processes live up to the highest standards of research integrity. We recognize that funders are many and varied in their scale, portfolio, disciplinary focus, and the extent to which they have procedures and governance arrangements to support research integrity. For all funders, adopting a RIPP will structure and coordinate research integrity practices, giving clarity and transparency to applicant institutions and researchers.

In this Consensus View, we highlight examples of best practice of funders worldwide to foster a research integrity culture. With these examples in mind, we suggest guidelines to support funders in taking a leading role in fostering research integrity. In so doing, we acknowledge the local contexts in which funders operate, but we believe that all funders, large and small, in all parts of the world, can and should contribute to improving research validity and building and maintaining trust in science through incentivising and mandating a culture of research integrity. Our core argument is that developing a tailored RIPP will contribute to building an institutional culture of research integrity, both within funding organizations and among the research organizations and individual researchers they fund. Based on empirical work from the SOPs4RI project, we have identified 6 key research integrity topics: researchers’ compliance with research integrity standards; expectations for research organizations; selection of grant applications; declaration of interests; monitoring funded research; and dealing with internal integrity breaches (Fig 1). We recommend that these topics should be included in a RIPP and provide guidelines on developing and implementing a RIPP.

**Fig 1:**
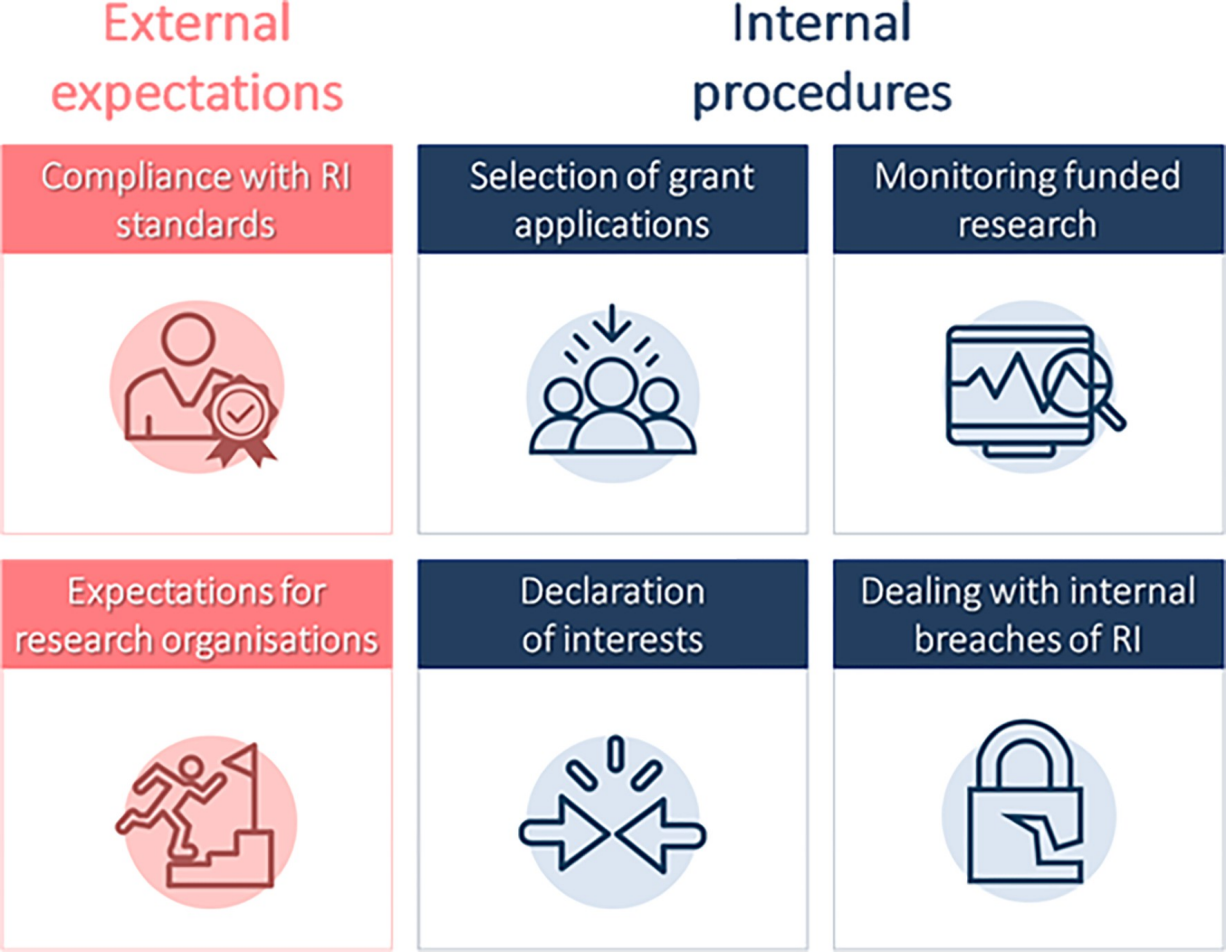
Topics to be covered in a RIPP for funders. An overview of the 6 most important topics identified by the SOPs4RI to be included in research funding organization’s RIPP. RIPP, Research Integrity Promotion Plan; SOPs4RI, Standard Operating Procedures for Research Integrity.

## Methodology

The recommendations in this article are based on the extensive empirical program applied in the context of the SOPs4RI project. In particular, we used a systematic literature review to obtain an overview of existing efforts to foster research integrity [12]. Subsequently, we engaged in consultations with key stakeholders within the funder community to actively solicit their views and interpretations of what constitutes a responsible research culture and how funding organizations, in their local and contextual capacities, can contribute to such a culture. These consultations were conducted using several methodological approaches, including Delphi surveys [13], expert interviews, focus groups [14], and co-creation workshops [15]. All these consultation efforts have built on each other, with the results of the Delphi study acting as the starting point for prioritizing the topics that were deemed most important for funders, and feeding into the interviews and focus group, which subsequently formed the basis of the co-creation workshops. The 6 topics described in the following section were finally agreed on after intensive deliberations of the author team, based on the empirical material gathered throughout our project. As a final step, we have set up an assessment system to prioritize initiatives according to a diverse group of stakeholders and project members. These initiatives are collected, divided under the 6 topics, and included in our online toolbox.

### Recommended topics to be addressed in a RIPP

**Fig 2:**
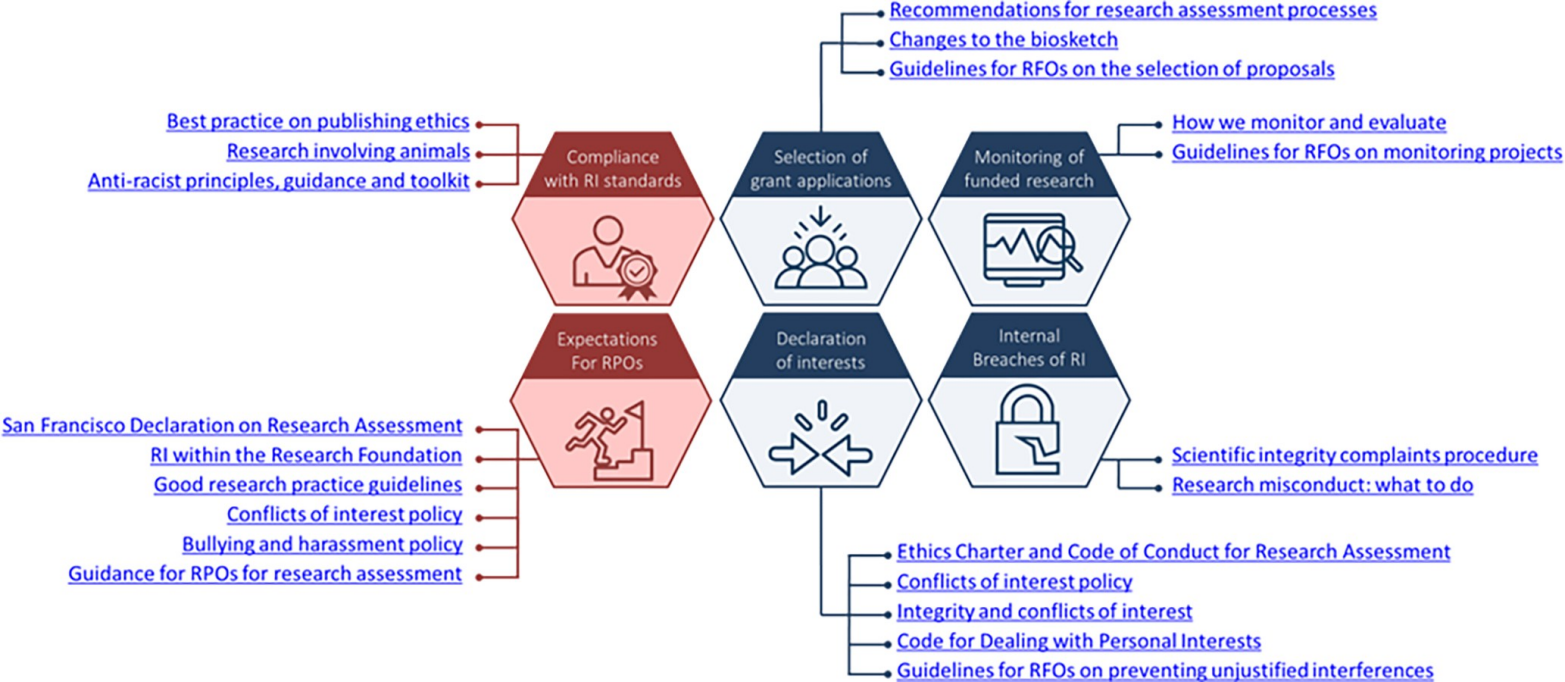
Currently implemented initiatives at funders to address the 6 RIPP topics. An overview of some of the initiatives described in this section, structured around the 6 key topics to be addressed in a RIPP. To find out more about the initiatives, please consult the references added in the main text. RFO, Research Funding Organisation; RIPP, Research Integrity Promotion Plan; RPO, Research Performing Organisation.

We recommend that funders develop and implement a RIPP that emphasizes the role of research integrity on 2 levels by setting out the expectations of applicants and research organizations and by providing guidance on their internal organizational procedures. Drawing on insights from our extensive empirical program, we have identified 6 pivotal topics for a funder’s RIPP (Fig 2).

In the following paragraphs, we describe the 6 topics and provide examples that illustrate how funders have adopted policies to support research integrity. These examples aim to inspire others to adapt these initiatives to their local context and thereby act as guiding lights to develop their own RIPP.

### Compliance with research integrity standards

We recommend that funders ensure that applicants for funding confirm their compliance with the current regulations and codes of conduct for research integrity. For example, the Danish Novo Nordisk Foundation outlines 15 principles [16] that should be adhered to by any applicant it funds. Such policies serve to raise awareness of research integrity in both the authoring of grant applications and the conduct of research. In a similar vein, in its 2009 guidelines on RCR, the US National Institutes of Health (NIH) [17] specifies that principal investigators applying for training grants and career development awards must submit a plan for RCR that includes face-to-face training for research staff and how they will incorporate RCR into daily practices. Designing such plans at the grant writing stage of doing research assists researchers in making responsible research practices part of their daily work. In addition, funders such as the Wellcome Trust [18] in the UK are increasingly demanding and enabling the researchers and organizations they fund to comply with standards of open science. This includes demands to publish open access and the creation of infrastructures to deposit study protocols.

### Expectations for research organizations

We recommend funders to, like the Research Foundation Flanders (FWO), mandate research performing organizations, as well as the researchers they employ, to follow a research integrity clause in grant contracts. Furthermore, organizations should meet other expectations including the provision of research integrity training and the monitoring of supervisors. As is now fairly common among research funders, this could also include mandates with respect to open science practices, including open access publishing, open data, data management plans, and requirements regarding preregistration or protocols for data analysis in research fields where this is appropriate. The requirements of the Canadian Tri-Agency Framework and the Horizon Europe program are prime examples of how funders can demand the implementation of research integrity practices in research-performing organizations. The FWO takes this to a next level by providing an innovative example of best practice in their desired profile [19] of potential applicants as well as the applicants’ host institutions. The profile describes in detail what FWO expects of a “good supervisor”.

We recommend that funders are also active in requesting detailed information on the research-performing organization’s RIPP and its implementation. The standards and requirements of such a RIPP for research organizations are outlined in our research organisation guideline [11] and follow the principles of the ECoC [1]. Funders should be informed by research organizations about how the research-performing organization deals with breaches of research integrity. When breaches occur, the funder should be informed promptly about allegations and the outcomes of the investigation by research organizations, or if required, instigate its own investigation.

### Criteria and processes for selecting grant applications

We recommend that the assessment and selection criteria for grant applications is transparent and publicly available. This relates both to the assessment of research proposals and applicants. To ensure optimal fairness in procedures and ultimately encourage integrity of the funded research, funders are expected to adhere to high standards of transparency in their evaluation process, as well as provide the right incentives in the criteria for judging applications. Regarding the former, the Norwegian Research Council [20] provides a particularly engaging example of achieving procedural transparency through short videos, infographics, and clear descriptions of every step of their assessment process. Regarding the latter, the US NIH’s BioSketch format of grant application [21], established in 2014, emphasizes the quality rather than quantity of published research in assessing applicants. More recently, the use of narrative CVs rather than publication lists and quantitative metrics is gaining support from funders including the Dutch Research Council (now) [22], the Health Research Board (HRB) in Ireland [23], and other signatories of the DORA declaration. These efforts are part of a movement towards responsible assessment criteria that can foster a culture of research integrity, which by now is supported by a substantial evidence base [24]. The Wellcome Trust [25] now restricts its funding to researchers at organizations that have Responsible Assessment Procedures in place. In addition, the Hong Kong Principles [26] describe 6 core principles to help assess researchers’ commitment to responsible research practices, and funders can use these principles to guide their approach to assessing applicants. Alternatively, several widely accepted statements have been released regarding elements to be avoided when evaluating research, including DORA [27] and the Leiden Manifesto [28]. These statements present a warning for the blind use of bibliometric or quantitative indicators, including h-indices, publication and citation counts, in the assessment of researchers and research organizations. Avoiding such purely quantitative assessments is important to allow for more holistic and qualitative evaluations of the merit of researchers and the work they produce.

### Declaration of interests

In 2020, the Fonds National de la Recherche Luxemburg (FNR) published an FNR Ethics Charter and Code of Conduct for Research Assessment [29], establishing a code of conduct for those involved in funding agencies. It stipulates how reviewers and the funder’s staff are expected to behave, with particular attention to impartiality and confidentiality. Similar to the FNR policy, we recommend that RIPPs for funders include procedures for the declaration of interests for funders’ internal staff, members of assessment and selection committees, and peer reviewers of submitted applications. All those involved in funding decisions should declare any financial, professional, or other interests that might be seen to influence a decision or to be affected by the outcome of a decision. Similar to the FNR, the Dutch funders NWO and ZonMw [30] have a set of guidelines relating to the declaration of interests, promoted by short movies and concrete descriptions. This can all help to create awareness of potential conflicts of interest and ways of dealing with them in an accessible way. In a slightly more legalistic fashion, the US NIH provides a statement on Integrity and confidentiality in Peer Review [31], outlining prohibitions for reviewers, including potential consequences.

### Monitoring funded research

We recommend funders to establish monitoring procedures, including the monitoring of responsible research practices for the research they fund, for example, including monitoring of open science and FAIR data practices. Most funders already have some monitoring procedures in place. For example, Ireland’s HRB sets out on its website [32] what it will monitor in a project through annual and final reports, as well as an end-of-grant evaluation survey. This includes submission of a Data Management Plan as a first output and making all research outputs openly available. Similarly, the Wellcome Trust [33] states that they will “consider whether researchers have managed and shared their research outputs in line with our requirements, as a critical part of the end-of-grant reporting process”. Through such monitoring processes and the transparency in communicating them, funders can guide researchers towards research and publication practices that foster research integrity. Simultaneously, we recommend researchers funders to try to avoid unnecessary administrative burdens on researchers, which are in fact considered to be potential drivers of questionable research practices [14].

For compliance with research integrity policies, the US NSF provides a good example of monitoring procedures. They conducted a review of levels of compliance with research integrity standards in a sample of 53 research-performing organizations, finding generally high compliance with applicable standards [6]. In their monitoring efforts, funders may distinguish between individual researchers, research consortia, and research organizations. Monitoring aspects could include compliance with good publication and dissemination practices, progress and alignment with the granted application, and the expenditure of funds (for example, to check for gross misuse of funds). While these monitoring endeavours can steer research practices into desirable directions, funders are also recommended to avoid creating an unnecessary bureaucratic burden on the researchers and organisations they fund, since this may be counterproductive [14].

### Dealing with internal breaches of research integrity

We recommend that funders have adequate and transparent procedures in place to manage potential breaches of research integrity standards by their staff, committee and panel members, and peer reviewers. Many funders already have such procedures in place, including established whistleblowing channels and protective mechanisms for both whistleblowers and the accused. The NWO operationalized this in an elaborate manner by setting up the NWO Scientific Integrity reporting center [34] to deal with cases of research integrity breaches both in the projects it funds as well as in its internal procedures. The center features confidential advisors and a scientific integrity committee and acts in accordance with the NWO Scientific Integrity Complaints Procedure and keeps close connections to the National Board for Research Integrity in the Netherlands. As such, it provides an inspiring example of how this part of a funder’s RIPP can be designed and implemented.

Large funding agencies could have a dedicated ombudsperson or an independent investigatory committee, whereas smaller funders could collaborate with other funders or national research integrity boards to make this feasible.

### Guidelines for developing and implementing a RIPP

**Fig 3:**
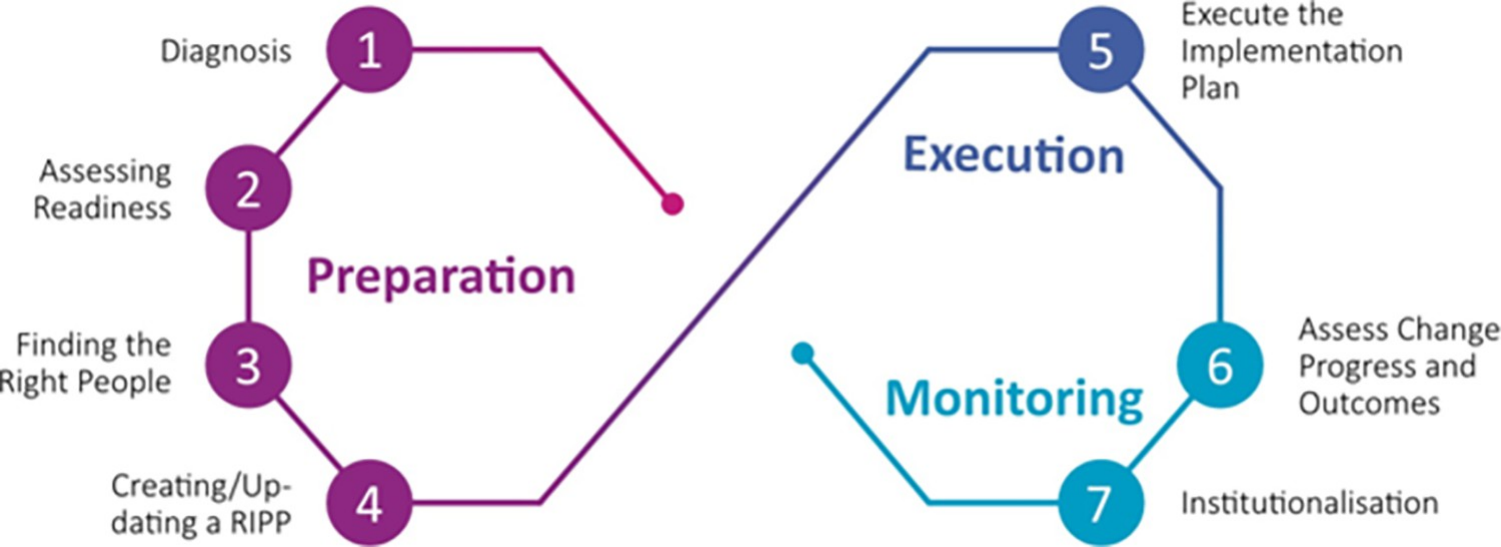
The implementation model. The model for RIPP implementation consists of 3 phases: Preparation, Execution, and Monitoring. Each of these phases involves tasks that are carried out in multiple steps. Importantly, the model proposes a cyclical format of creating, maintaining, and revising a culture of integrity. RIPP, Research Integrity Promotion Plan.

To assist research funders in designing a RIPP and implementing concrete actions that will foster a culture of research integrity, we have created Implementation Guidelines and a RIPP Template that can be accessed online. The guidance to create and implement a RIPP follows the model depicted in Fig 3, which is recommended to be executed in a cyclical manner.

As input to the first cycle, a funding agency may use the aforementioned 6 topics to be addressed in a RIPP. The first cycle aims to create and implement a first draft of the RIPP. This RIPP will then be the input for the next cycle. Each cycle consists of 7 distinct steps: diagnosis, assessing readiness, finding the right people, creating/updating a RIPP, executing the implementation plan, assessing change progress and outcomes, and institutionalization (Box 1).

**Box 1. Seven steps for developing and implementing a RIPP**

**Diagnosis**

Gather information to assist in the diagnosis of what change is needed: Which of the RIPP topics are most relevant to the organisation and require the most attention? This also could also include setting an aspirational marker on the horizon, a vision to work towards.

**Assessing readiness**

Assess the readiness of the organization for change, particularly assessing the resource capacity and willingness of the organization to take on the demands that effective change requires. This includes, among others, senior leadership’s capability to guide change, the availability of sufficient resources, and the preparedness for change among the organisation’s members.

**Finding the right people**

Identify the right people to promote and execute the process, forming a *change coalition*. A change coalition consists of change agents and role models from diverse organizational units that will guide the creation and implementation of a RIPP. Important aspects to take into account comprise the potential need for specific training or preparation for the identified people; inclusion of all relevant types of staff: for example, junior and senior researchers, mid-level management, and people centrally placed in the organisation’s social network and with the right characteristics: trustworthy, supportive, and honest.

**Creating/updating a RIPP**

Write the RIPP, specifying goals, relevant stakeholders, the required organizational setup, relevant tools, specific actions and responsibilities, and a set of indicators or targets to monitor effectiveness. Specific guidelines, recommendations, and examples from the SOPs4RI toolbox can be used for inspiration or implementation.

**Executing the implementation plan**

Properly inform all stakeholders and roll out the change plan. In case the intended change involves a major restructuring of (some) stakeholders’ daily workflow, we suggest considering a gradual change process. A gradual process can include several pilot tests, experiments, and local initiatives, which together make complex change easier to implement. At this stage, it is also crucial to allow change recipients to provide feedback and make local adjustments to broader change plans.

**Assessing change progress and outcomes**

Perform periodic assessments based on the predetermined set of change indicators to verify whether the planned change is producing anticipated outcomes and/or unintended side effects. This also includes an evaluation of the resources required.

**Institutionalization**

Integrate the novel procedures into the funder’s workflow, culture and operating systems. Based on the assessment, the RIPP is revisited, resources and responsibilities are allocated for long-term implementation, and the change coalition’s relevant experiences from their organizational unit are implemented into the procedures and policies of the entire organization.

For more details and guidance on each step, we refer readers to the Implementation Guidelines created by the SOPs4RI consortium [35]. Some of the 6 topics in a RIPP and some of the 7 steps to create and implement a RIPP may be of greater or lesser relevance to a funder’s local context. The RIPP template and implementation guidelines are therefore designed to be used flexibly and tailored to the user’s local context and can be used by both organizations that already have research integrity policies in place and organizations that are about to start on their research integrity journey.

Based on the monitoring of one cycle, a new diagnosis of the next cycle can be readily performed. We recommend repeating cycles at regular intervals: at least every 3 or 4 years. To avoid additional or redundant administrative workload, we suggest the cycles be coupled with existing evaluation cycles already taking place regularly, such as external or internal audits. Integrating the continuous efforts on the RIPP with existing procedures might both reduce administrative burden and allow research integrity to become an integral aspect of the organization’s policies and workflow.

### Time to follow suit

The global research community has seen a considerable increase in attention on research integrity. Researchers across the sciences, social sciences, and humanities and their funders, large and small, must be, and increasingly are, committed to supporting a culture of research integrity. The SOPs4RI project has found examples in many countries of funders that are picking up the challenges posed by research integrity. We are confident that this movement will gather pace over the coming years, supported by contributions made by funders.

However, we also recognize and acknowledge that we have a long way to go. Best practice initiatives are scattered, fragmented, many are only recently implemented, and implementation often turns out to be challenging. In addition, not all funders are equally well positioned and resourced to enable them to implement all the recommendations and best practices we outline in this Consensus View. We acknowledge that it takes time to change cultures, and we recognize that funders are varied in their aims and the extent to which they have resources available for procedures and governance arrangements to support research integrity. We would also like to emphasize that successful implementation of RIPPs is dependent on local contexts and should take disciplinary differences into account, as successful implementation depends on how practices are taken up by a funder’s main stakeholders. Also, research integrity matters do not stand on their own, but rather are related to several other discussions. Reflecting this, funders on their own and collectively are aiming to connect their approaches to addressing related topics; for example, Science Europe have established several working groups on research assessment, open access (publications), open data, and research culture [36].

The SOPs4RI toolbox has been specifically developed to assist funders worldwide in their endeavors to implement research integrity policies and procedures. We believe that this toolbox, consisting of research integrity guidance on all 6 topics, Implementation Guidelines, and a RIPP Template, is an important step in assisting funders to achieve this for their own organization and for the researchers and research organizations they fund. The toolbox can also assist in creating some common standards across funders, leaving enough flexibility for local adaptations while harmonizing requirements across different contexts.

An additional challenge for the implementation of new policies and procedures is the consistent lack of evidence about their effectiveness and wider consequences. Part of the shortage of empirical data on these topics is due to the relatively recent nature of new initiatives and hence the absence of sufficient time to evaluate them. But there are other reasons too. While there has been a drastic shift towards openness and transparency in many aspects of academic practices, for example, journal articles, peer review reports, and research data, the processes at research funding organizations remain largely opaque and unexamined. This lack of evidence creates serious challenges for the research community to properly assess the merits of diverse funding practices and selection procedures. Therefore, in addition to our call to funders to establish a RIPP along the lines described in this Consensus View, we urgently plea for them to follow the global transition towards more transparency in research by collecting and making data on their practices openly available, with the aim to improve them. Particularly, we encourage funders to be more transparent about their criteria for evaluating proposals, the methods used to select proposals to be funded, and funders’ efforts to avoid or deal with internal breaches of research integrity standards. Among other areas, transparency about these aspects will allow the research community to understand funders’ processes. This should be part of holistic attempts to address research integrity challenges. Only efforts fostering research integrity cultures in their broad conception are likely to be effective.

In addition to establishing a RIPP, funders can also contribute to research integrity by funding or facilitating research into responsible research or breaches thereof. Such efforts could include the direct funding of studies into research integrity, direct funding of research integrity training, or funding of core facilities that can help with experimental design, research reporting, and data analysis for individual researchers. This will ultimately contribute to a better understanding of how we can collectively build a research culture that is conducive to research integrity.

In this Consensus View, we provided an overview of initiatives that are currently changing the funding landscape towards one that is more conducive to research integrity. Through empirical work, we identified 6 topics that are crucial in fostering a culture of research integrity in funding organizations and have provided guidelines to develop and implement a RIPP. Several funders have taken the first important step by emphasizing the importance of research integrity, requiring that research organizations should have research integrity policies in place, and organizing their own procedures to foster research integrity. We herald these front runners, and we believe that now is the time for more funders to take up the challenge to follow suit.

## Abbreviations

EC: European Commission
ECoC: European Code of Conduct for Research Integrity
FNR: Fonds National de la Recherche Luxemburg
HRB: Health Research Board
NIH: National Institutes of Health
NSF: National Science Foundation
RCR: Responsible Conduct of Research
RIPP: Research Integrity Promotion Plan
SOPs4RI: Standard Operating Procedures for Research Integrity
